# Transdiagnostic profiles of socio‐affective functioning in adolescents at‐risk of poor mental health

**DOI:** 10.1002/jcv2.70137

**Published:** 2026-06-22

**Authors:** Alex Lloyd, Duncan Astle, Tom (Chin‐Han) Wu, Nikolaus Steinbeis, Ritika Chokhani, Laura Lucas, Pasco Fearon, Essi Viding

**Affiliations:** ^1^ Clinical, Educational and Health Psychology, Psychology and Language Sciences University College London London UK; ^2^ MRC Cognition and Brain Sciences Unit University of Cambridge Cambridge UK; ^3^ Centre for Family and Adolescent Research, Department of Psychology University of Cambridge Cambridge UK

**Keywords:** adolescence, emotion processing, machine learning, social relationships, transdiagnostic

## Abstract

**Background:**

Adolescence is a developmental period during which many mental health problems emerge or worsen. Recently, there has been a shift towards identifying risk factors that predict psychopathology across a range of diagnostic boundaries—known as ‘transdiagnostic’ approaches. There are several social and emotional risk factors for specific mental health diagnoses that have been well‐characterised. Yet, there is limited understanding about how these socio‐affective risk factors map onto *transdiagnostic* symptoms. We identified profiles of socio‐affective functioning in early adolescence and how these profiles are associated with transdiagnostic symptoms of mental health problems.

**Methods:**

Adolescents at heightened risk of general psychopathology aged 12–14 (*N* = 559, *M*_age_ = 13.26, SD_age_ = 0.72, 58.14% female) were recruited as part of a mental health trial. Participants completed questionnaire and task‐based measures assessing emotion functioning (emotion perception, emotion regulation, and interoception), social relationships (with peers and family members), and mental health. Using a simple artificial neural network that projects a high‐dimensional input to a 2D topology we were able to map differences in questionnaire‐ and task‐based socio‐affective profiles. Clustering was then used to identify zones within that 2D topology, indicating relatively homogenous profiles. Associations between these zones and transdiagnostic mental health symptoms were examined.

**Results:**

We identified three reliable clusters of socio‐affective functioning from the questionnaire measures and four clusters from the task‐based measures. There were significant differences between questionnaire clusters on general psychopathology and p‐free internalising symptoms, whereas there were only significant differences between task clusters on general psychopathology.

**Conclusion:**

This study demonstrated the potential of data‐driven methods to derive profiles of socio‐affective functioning that are associated with transdiagnostic mental health problems. Through identifying socio‐affective mechanisms that characterise these clusters, these findings can be used to identify active ingredients for future intervention development to prevent the onset and worsening of mental health problems in adolescence.

## Key Points

**What's known?**

- There are well‐known social and emotional processes in specific mental health problems.

**What's new?**

- This study uses data driven methods to identify social and emotional (‘socio‐affective’) clusters of functioning.
- The clusters derived from these analyses identified groupings at different levels of symptom severity, but did not align with traditional diagnostic boundaries.

**What's relevant?**

- These socio‐affective profiles highlight the bidirectional influence of social and emotional processes in adolescents at heightened risk for psychopathology and may be promising targets for future intervention.

## INTRODUCTION

Adolescence is a period of significant physical, social and neurocognitive development (Spear, 2000). This developmental period is also marked by an increased susceptibility to the onset of mental health problems, with the median age for the onset of all mental health problems occurring at 14.5 years (Solmi et al., 2022). Within adolescent populations, the prevalence of mental health problems is also increasing (Dykxhoorn et al., 2024), motivating investigation into social and emotional (hereafter ‘socio‐affective’) processes associated with psychopathology. While several socio‐affective processes are well‐characterised in specific diagnoses, such as maladaptive emotion regulation in young people with depressive disorders (Schäfer et al., 2017), there is limited understanding about how these socio‐affective processes relate to *transdiagnostic* symptoms. Transdiagnostic symptoms are those which commonly present in and contribute to the maintenance of mental health problems across traditional diagnostic categories. For example, rumination is one transdiagnostic symptom common across depression, anxiety, bipolar and Obsessive‐Compulsive Disorder. Further, socio‐affective processes are typically studied in isolation and there is limited understanding about how socio‐affective processes may cluster together to increase susceptibility to mental health problems. To address these gaps, this paper used data‐driven methods to identify socio‐affective profiles in a sample of adolescents at heightened risk for psychopathology and examined how these profiles were associated with transdiagnostic mental health outcomes.

Recent approaches to the study of psychopathology have moved away from nosology and increasingly adopted a ‘transdiagnostic’ framework, which have raised questions about the validity of diagnostic boundaries (Dalgleish et al., 2020; Kreuger & Eaton, 2015). According to some transdiagnostic approaches, an individual's susceptibility to experiencing any mental health problem is at least partly predicted by a general liability or latent variable named the ‘p‐factor’ (Caspi et al., 2014; Fusar‐Poli et al., 2019). Most transdiagnostic models are organised hierarchically, with separate subdomains of symptoms of psychopathology reflecting ‘internalising symptoms’, such as those typically exhibited in mood and anxiety disorders, and ‘externalising symptoms’, such as those typically exhibited in conduct and substance misuse problems (Kreuger & Eaton, 2015). Transdiagnostic models of psychopathology have demonstrated good fit to the distribution of mental health symptoms in adolescent populations (e.g., Patalay et al., 2015) and can explain the high co‐occurrence of mental health problems in adolescent and adult populations (Solmi et al., 2023). Additionally, transdiagnostic models tie in with evidence that diagnostic categories contain heterogeneous groups of individuals, which is why the models focus on symptoms rather than diagnostic labels (Nolen‐Hoeksema & Watkins, 2011; however, see Watts et al., 2024 for a critical review of general factors of psychopathology).

There is already good evidence for several candidate mechanisms that are transdiagnostic risk factors for mental health problems in young people. For example, the quality and quantity of social relationships during adolescence predict mental health problems across internalising and externalising symptoms (Mitic et al., 2021). Indeed, the association between social relationships and psychopathology may create a ‘vicious cycle’, whereby poorer social relationships exacerbate mental health problems which, in turn, lead to further breakdown of social relationships (Klinck et al., 2020; Wu et al., 2025). In addition, individual differences in emotion processing—including emotion perception, emotion regulation, and interoception—are associated with mental ill‐health during adolescence (McLaughlin et al., 2015; Murphy et al., 2017; Silvers, 2022). For example, poorer emotion regulation abilities are associated with internalising symptoms that characterise mood disorders (Henry et al., 2016) and with externalising symptoms such as conduct problems (Herpertz et al., 2008). These data suggest that there are socio‐affective processes that contribute to symptom risk across diagnostic boundaries and may therefore be associated with an individual's general likelihood of experiencing psychopathology.

Despite existing evidence that social relationships and emotion processing relate to mental health problems across symptom profiles, there is still limited research that has examined how these processes act *in combination* to predict mental health outcomes. This is notable, as social relationships and emotion processing abilities have a bidirectional relationship with one another (Raza et al., 2024). For example, higher emotion regulation predicts greater family connection in early adolescence (Demkowicz et al., 2024) and there is evidence that emotion regulation and social information processing interact to predict externalising behaviours (Smeijers et al., 2020).

In this context, methods from machine learning may be especially potent as a data‐driven approach to identify profiles across a range of input measures. In particular, unsupervised machine learning methods can find meaningful subgroups in a population and are an unbiased approach towards categorising profiles. Self organising maps (SOMs) are a particular form of unsupervised machine learning that summarise high dimensional data on a 2D grid. The grid visualises nodes representing the input measures, where nodes closer to one another are more similar on input measures compared to nodes further apart (Antonucci et al., 2025). The advantage of SOMs, relative to other methods (e.g., latent profile analysis, principal component analysis), are that these maps can be flexible to the structure of input data (i.e., allow for non‐linear relationships) and allow for better separation of classes in cluster analyses of complex data (Hulle, 2000), which is particularly advantageous for task‐based measures, which often do not have linear relationships to measures collected via other modalities. In addition, as SOMs are visualised on grids, applying and visualising cluster analyses of these maps provides an intuitive way to interpret relationships between classes.

Work using SOMs has identified profiles of executive functioning, cognitive and language abilities in young people with neurodevelopmental disorders (Mareva et al., 2023, 2024). Notably, profiles were uniquely related to mental health and educational outcomes, highlighting important heterogeneity in this population. While data‐driven clustering analyses have previously been applied to characterise profiles within diagnostic categories (e.g., socio‐affective profiles in depression or cognitive subtypes in psychosis; Chan et al., 2023; Green et al., 2020), there is limited work that has examined how interactions between socio‐affective processes are associated with transdiagnostic outcomes.

The aim of the present paper was to apply a data‐driven approach to identify profiles of socio‐affective functioning that were associated with transdiagnostic mental health outcomes in young people at heightened risk for psychopathology. Unlike previous work (e.g., Chan et al., 2023), this study used a transdiagnostic framework to contribute to our understanding of how socio‐affective processes are associated with symptoms of psychopathology. These findings can contribute to developing tailored support for young people at risk of poor mental health by targeting socio‐affective domains that predict transdiagnostic symptoms. The research question for this study was whether there are socio‐affective profiles that are associated with transdiagnostic symptoms in adolescence. Given the data‐driven clustering method used, we do not have predictions for specific profiles that will be identified from the data and therefore take an exploratory approach to the identification of clusters and their relationship to transdiagnostic mental health problems.

## METHODS

### Participants

Participants for this study were part of a randomised controlled intervention trial: Building Resilience through Socioemotional Training (ReSET; see Viding et al., 2024 for a protocol; trial registry: ISRCTN88585916). The sample consisted of adolescents aged 12–14 recruited from mainstream schools in London and the South East of England. Participating schools had a minimum of 30% of students eligible for free school meals, indicating socioeconomic diversity. Participants were identified as being at risk for poor mental health through the self‐report version of the Strengths and Difficulty Questionnaire (SDQ; Goodman, 1997), which was administered to Years 8 and 9 at participating schools. Eligible adolescents were those scoring 15 or above on the SDQ total difficulties score, determined from nationally representative data to be in the top 25th percentile for this age group (University of Essex, 2022). There were no other exclusion criteria, though Special Educational Needs and Disabilities (SEND) diagnoses were recorded, which accounted for 20.57% of the sample.

The sample consisted of 559 participants (*M*_age_ = 13.26, SD_age_ = 0.72, 58.14% female) and was diverse in ethnicity (see Table 1 for a summary of demographic characteristics). Participants and their parents/carers provided informed consent prior to taking part. Participants were remunerated £15 in vouchers for completing the assessment battery. This study was approved by UCL's research ethics committee (ref: 21815/001).

**TABLE 1:** Demographic characteristics of participants.

Participant demographics	
Age, mean (SD)	13.26 (0.72)
Gender	
Male	220 (39.36%)
Female	325 (58.14%)
Other	7 (1.25%)
Prefer not to say	7 (1.25%)
Ethnicity	
English/Welsh/Scottish/Northern Irish	171 (30.59)
Irish	2 (0.36%)
Any other white background	48 (8.59%)
White and Black Caribbean	10 (1.79%)
White and Black African	13 (2.33%)
White and Asian	10 (1.79%)
Any other mixed/multiple ethnic background	19 (3.40%)
Indian	66 (11.81%)
Pakistani	19 (3.40%)
Bangladeshi	22 (3.94%)
Chinese	3 (0.54%)
Any other Asian background	36 (6.44%)
African	38 (6.80%)
Caribbean	17 (3.04%)
Other Black/African/Caribbean background	13 (2.33%)
Arab	19 (3.40%)
Other	21 (3.76%)
Sexual orientation	
Completely heterosexual/straight	379 (67.80%)
Mainly heterosexual/straight	39 (6.98%)
Bisexual	45 (8.05%)
Mainly gay or lesbian	2 (0.36%)
Don't know	6 (1.07%)
Prefer not to say	55 (9.84%)
Other	34 (6.08%)

### Measures

Participants completed a battery of questionnaire and task‐based measures. Measures were selected in the trial to capture mechanisms that might mediate the efficacy of the trial, rather than for their ability to capture the cluster structure optimally. Questionnaire and task measures were selected to capture similar processes (specifically emotion regulation and emotion perception) through different modalities. All measures were administered by a trained researcher via an iPad device and took approximately 1.5 h to complete. The Cronbach's alpha values reported below refer to values calculated in the current dataset.

#### Emotion regulation

Emotion regulation was measured by one questionnaire measure and two task‐based measures. The Emotion Regulation Questionnaire for Children and Adolescents is a questionnaire measuring the use of reappraisal (Cronbach's *α* = 0.83) and suppression (Cronbach's *α* = 0.64) emotion regulation strategies (Gullone & Taffe, 2012). Emotion regulation was further measured using two tasks: The first task presented negatively‐valenced interpersonal scenarios (e.g., ‘Having an argument with a friend’) and measured participants' ability to regulate the negative emotions elicited from that scenario. Emotion regulation ability was operationalised by computing the difference in self‐rated affect between trials in which the participant was asked to passively imagine the scenario or actively downregulate their emotions (see Greimel et al., 2020 for a schematic outline of this task). The second task was identical, with the exception that pictures eliciting negative affect were presented instead of scenarios. These images were selected from the International Affective Picture System stimuli set and were restricted to those that had been used with developmental populations in previous studies (Sharp et al., 2006; Vink et al., 2014).

#### Emotion perception

Emotion perception was measured by one questionnaire and two task‐based measures that assessed biases in appraisals of emotional experiences. Self‐reported emotion perception was measured using the Attributional Styles Questionnaire, which yields two subscales: Positive (Cronbach's *α* = 0.49) and negative attributions (Cronbach's *α* = 0.49; Peterson et al., 1982). Emotion perception was further indexed using the emotional intensity morphing task (Bland et al., 2016). The task yields a ‘point of detection’, which is participants' sensitivity towards recognising emotion in others. Specifically, the task requires participants to view a neutral face, and respond once they identify an emotion, or view an expressive face and respond once they no longer identify the emotion. Participants' responses are coded such that higher values indicate less sensitivity. The interpretation bias task was used to measure individual differences in hostile attributions (Penton‐Voak et al., 2012). In this task, participants are presented with a face displaying an ambiguous face and asked to identify the emotion as either happy or angry, which is used to identify the proportion of faces participants perceive as hostile. Two variables were derived from this task: The first is the participant's balance point, where higher values indicate a higher proportion of positive attributions, and the second is participants' match point, where higher values indicate greater consistency in their attributions. The match point is a measure of volatility in participants' attributions, irrespective of their bias for positive or hostile attributions.

#### Interoception

Interoception was measured using two age‐appropriate scales assessing interoceptive accuracy (Cronbach's *α* = 0.90; Murphy et al., 2020) and interoceptive attention (Cronbach's *α* = 0.91; Gabriele et al., 2020). Each measure had 20 items on a 5‐point scale.

#### Social relationships

Social relationships were measured for caregivers and peer relationships. To measure caregiver relationships, participants completed two measures: the inventory of parent and peer attachment (IPPA; Armsden et al., 1989) for their primary female (Cronbach's *α* = 0.93) and male caregiver (Cronbach's *α* = 0.95). Participants also completed five self‐report measures of peer relationships: the IPPA for peers (Cronbach's *α* = 0.89; Armsden et al., 1989), the multidimensional peer victimisation scale (Cronbach's *α* = 0.93; Mynard & Joseph, 2000), the UCLA loneliness scale (Russell et al., 1980), a single item measuring the number of individuals the participant seeks advice from, and three items from the Social Network Analysis of Risky Behaviours in Early Adolescence battery, which probes one's self‐perception relative to peers (Franken et al., 2016). In addition, two social network items were also collected during the screening process used to identify eligible participants. These social network measures allowed us to derive two ‘indegree’ measures, which indicated the number of times a participant was nominated by their peer in response to two questions: ‘Who is your best friend?’ and ‘Who do you like?’ (de Vries et al., 2021).

We included several additional variables known to interact with socio‐affective processes in predicting mental health outcomes.

#### Substance misuse

Substance misuse was measured using the Alcohol Use Disorder Test (AUDIT; Bush et al., 1998) and drug use with the Drug Use Disorder Test (DUDIT; Voluse et al., 2012). As the prevalence of alcohol and drug use was low in this sample, we opted to take the first item of these scales which measures the frequency of either alcohol or drug use (ISRCTN88585916).

#### Insomnia

Insomnia was calculated through bespoke items measuring the frequency and impairment of sleep disturbances, each reported on a 7‐point scale. Insomnia cases were defined as those that report both a frequency of ≥3 and impairment of ≥3 for sleep disturbances on these two items. This variable was subsequently entered as a single binary variable into the analysis.

#### Academic pressure

Academic pressure was measured with an 8‐item questionnaire. The items assess self‐perceived pressure from the participant and those around them to achieve academic success, with higher values indicating the individual feels more academic pressure. This novel scale demonstrated good reliability (Cronbach's *α* = 0.79).

#### Working memory

Participants completed a computerised version of the backwards digit span, which presents participants with numbers which they are required to enter in reverse order. This task estimates participants' digit span threshold, with higher values denoting greater working memory capacity (Dumontheil et al., 2020). The inclusion of working memory was motivated by evidence that it may be a transdiagnostic risk factor for psychopathology (Huang‐Pollock et al., 2017) but also one that predicts emotion regulation (Barkus, 2020) and may therefore interact with emotion processing abilities to predict mental health outcomes.

#### Mental health

Transdiagnostic mental health outcomes were measured using: The Strengths and Difficulties Questionnaire (Cronbach's *α* = 0.75; Goodman, 1997) and the Me and My Feelings Questionnaire (Deighton et al., 2014). To note, the SDQ data was collected for a second time during after participants had been identified as eligible and enrolled into the trial. As specified in the project protocol (ISRCTN88585916), these scales were used to derive a latent bifactor model of psychopathology, which consisted of a general psychopathology factor (p‐factor) and the specific factors of internalising symptoms and externalising symptoms (Patalay et al., 2016; for factor loadings see Supporting Information S1: Appendix S1 and Table S1). Factor scores were then used as outcome variables in the analyses. Further, wellbeing was measured using the Warwick and Edinburgh Wellbeing Scale (Cronbach's *α* = 0.85; Tennant et al., 2007).

### Analysis plan

#### Missing data and data transformation

Missing mental health data (SDQ, M&MF, and WEMWBS) was estimated using mean imputation for cases where fewer than 20% of the data were missing for that scale; there are no cases where greater than 20% of data were missing for a subscale. Mean imputation is an imputation method whereby the average of the present items is calculated and used to impute the value of the missing item(s). This imputation method was pre‐registered (ISRCTN88585916) and used in this study to maintain consistency with the main trial. Only measures collected after participants had been enrolled into the trial were imputed (i.e., initial SDQ scores used to identify participants were not imputed). For all other measures, excluding the IPPA for male and female caregivers, missing data were imputed using the MICE package for R (van Buuren & Groothuis‐Oudshorn, 2011). The IPPA measures were excluded from multiple imputation as participants without a primary male or female caregiver were not required to complete this measure, and therefore missing data was meaningful. For measures that were imputed, we used the predictive mean matching method to impute 20 datasets over 60 iterations. All questionnaire‐ and task‐based variables within the dataset were used as auxiliary variables when imputing the data, with the exception of participants' ID and a variable denoting the academic term they completed the measures. Participants' school ID was set as a grouping variable. A random dataset was selected from those imputed to be used in the cluster analysis (as has been done for other methods without standardised imputation methods; McElroy et al., 2018). Given the small percentage of missing data, there was minimal variation in variable means between the 20 imputed datasets, reducing the likelihood that variations between datasets would influence the SOM or cluster analyses. Prior to the SOMs and cluster analyses, data were z‐transformed to avoid measures with larger variance having disproportionate weight in the SOMs (Gao et al., 2023). Variables were standardised within the full sample using the scale() function for R.

#### Self‐organising maps and cluster analysis

Given evidence that questionnaire and task‐based measures assess distinct dimensions (Friedman & Banich, 2019), we decided that SOM and cluster analyses would be conducted separately for questionnaire and task‐based measures. This decision was further motivated by evidence that the modality of assessment (i.e., self‐report vs. behavioural) can bias clustering solutions, such that questionnaire and task‐based measures artificially cluster together rather than representing statistically or conceptually distinct profiles (Gao et al., 2023).

SOMs are a type of unsupervised artificial neural network used to reduce dimensionality and identify structure in complex datasets (Wehrens & Buydens, 2007). SOMs do not assume a specific number of latent dimensions within a dataset but rather map data onto a fixed grid of nodes that preserve topological relationships. Each node is represented by a prototype vector (i.e., weight vector), which describes how that node relates to the input features. For each input vector (i.e., set of participant scores), the SOM algorithm will search for the node with the weight vector closest to the input vector (minimising distance as measured using Euclidean distance). Weights will be updated with each search until the SOM algorithm converges on a map that best reflects the structure of the input data, which is presented on a 2D grid. In cases with missing data, the kohonen R package we used to implement the SOMs (Wehrens & Buydens, 2007) only updates non‐missing dimensions of the weight vectors. When creating the final map, incomplete data are mapped to the Best Matching Unit based on available input data. The result of the algorithm is that participants are assigned to nodes based on similarities in their input performance (i.e., scores on the questionnaires or task‐based measures). Nodes with similar weight profiles are positioned closer together on the map and are used as the basis for the clustering analysis (Wehrens & Buydens, 2007).

We implemented SOMs in a two‐stage process, first conducting a training stage to globally organise the map followed by a second fine‐tuning stage search to refine the weights of each node. Both the training and fine‐tuning phase ran on a 15 × 15 grid using the default (‘bubble’) neighbourhood function (Wehrens & Buydens, 2007). However, there were several parameters that were altered between these two phases. Specifically, in the training phase, we used learning rates from 0.03 to 0.005, had a neighbour radius from 5 to 2, and ran for 200 iterations. In the fine‐tuning phase, the learning rate was lowered from 0.005 to 0.0005, the neighbourhood radius was reduced from 2 to 0.5 and there were 1000 iterations. The questionnaire‐based SOM had had 19 features, and the task‐based SOM had 6 features. Both questionnaire and task‐based SOMs explained a good proportion of the variance in the data (>70%) and had acceptable topographic error (<0.15), suggesting the maps were able to effectively capture the structure of the data.

K‐means clustering was then used on the nodal weights to derive the optimal number of clusters on the map. We used the R package NbClust (Charrad et al., 2014) to identify the solution that best represented the data based on a consensus approach across 30 indices. This analysis provides each participant with a cluster assignment based on the nodes they were assigned to in the SOM, which was plotted on the original map to inspect whether the clusters were able to distinguish between participants with similarly weighted nodes (Bathelt et al., 2018; Dalmaijer et al., 2022; Mareva et al., 2024).

Silhouette scores, which indicate the statistical discriminability of clusters, were generally low for the questionnaire‐based clusters (*M* = 0.06). However, as an alternative measure of the reliability of clusters, we conducted bootstrapped the cluster analysis for 100 reps to indicate the stability of cluster membership. These bootstrap analyses provide Jaccard similarity scores, which indicate how stable cluster assignment is across samples, with values of <0.6 indicating poor stability, scores between 0.6 and 0.75 indicating moderate stability, and scores of >0.75 indicating high stability (Zumel & Mount, 2014). For the questionnaire‐based clusters, two clusters had moderate Jaccard similarity scores (0.70 and 0.75, respectively), which indicate the clusters are measuring a meaningful pattern in the data (Tang et al., 2021). In contrast, one cluster had a poor Jaccard similarity score of 0.47, which can suggest the cluster is unstable. Applying the same analyses to the task‐based clusters, the average silhouette score was 0.12. Assessing the stability of clusters, two clusters had moderate stability (0.6 and 0.78) whereas a further two had poor stability (0.55 and 0.44). The low silhouette scores suggest caution is needed when drawing conclusions about the boundaries between clusters in both sets of analyses.

#### Cluster characterisation and comparisons

To compare performance on the questionnaire and task‐based measures between clusters, *t*‐tests corrected for false discovery rates (FDR) were conducted, which allowed us to characterise the clusters based on socio‐affective variables. The FDR correction was applied to this analysis given the relatively large number of comparisons in analyses characterising the socio‐affective profiles of each cluster. To test whether these clusters could explain differences in transdiagnostic mental health measures, we conducted one‐way Analysis of Variance (ANOVA) analyses with cluster membership entered as the independent variable and raw scores on mental health measures (SDQ total difficulties, WEMWBS total score) and factor scores for the p‐factor (p‐factor, p‐free internalising problems and p‐free externalising problems) entered as the dependent variables in separate models. To account for the multiple comparisons across mental health outcomes, we apply the FDR correction to the ANOVAs for each conceptual family of hypotheses (i.e., how the questionnaire‐ and task‐based clusters related to mental health outcomes). Post‐hoc tests were conducted for significant main effects using the Bonferroni correction for multiple comparisons.

## RESULTS

### SOMs and clustering

**FIGURE 1:**
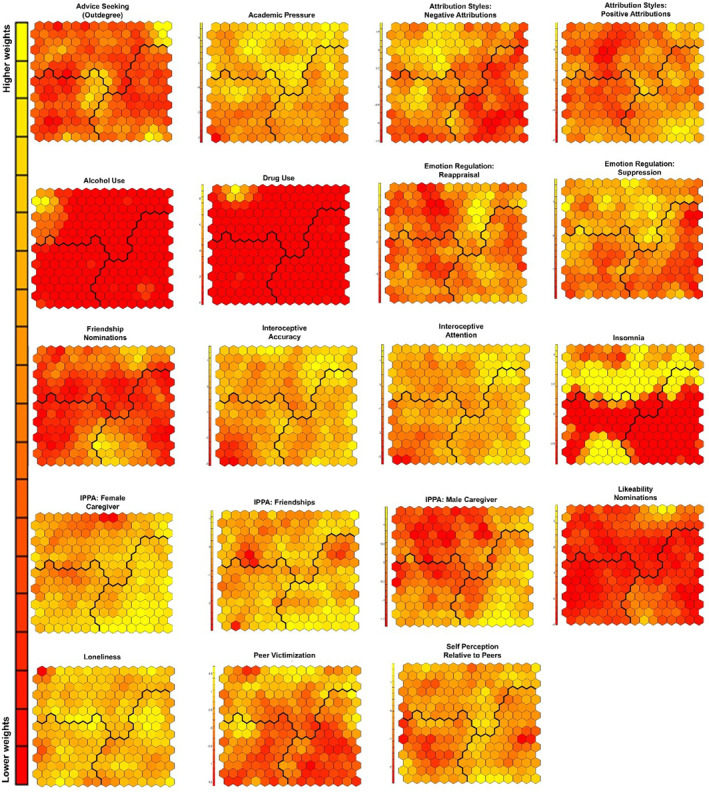
Nodal weight distributions from the SOMs, split by questionnaire measure. For each measure, hexagons represent weights of that measure, with red hexagons representing low weights (i.e., lower scores) and yellow hexagons representing high weights (i.e., higher scores). Boundaries depicted by the black line indicate borders between the data‐driven clusters applied to the SOM. Locations of the nodes on the *x*‐axis and *y*‐axis are determined by similarity to neighbouring node rather than denoting specific values on input measures. SOM = self‐organising maps.

**FIGURE 2:**
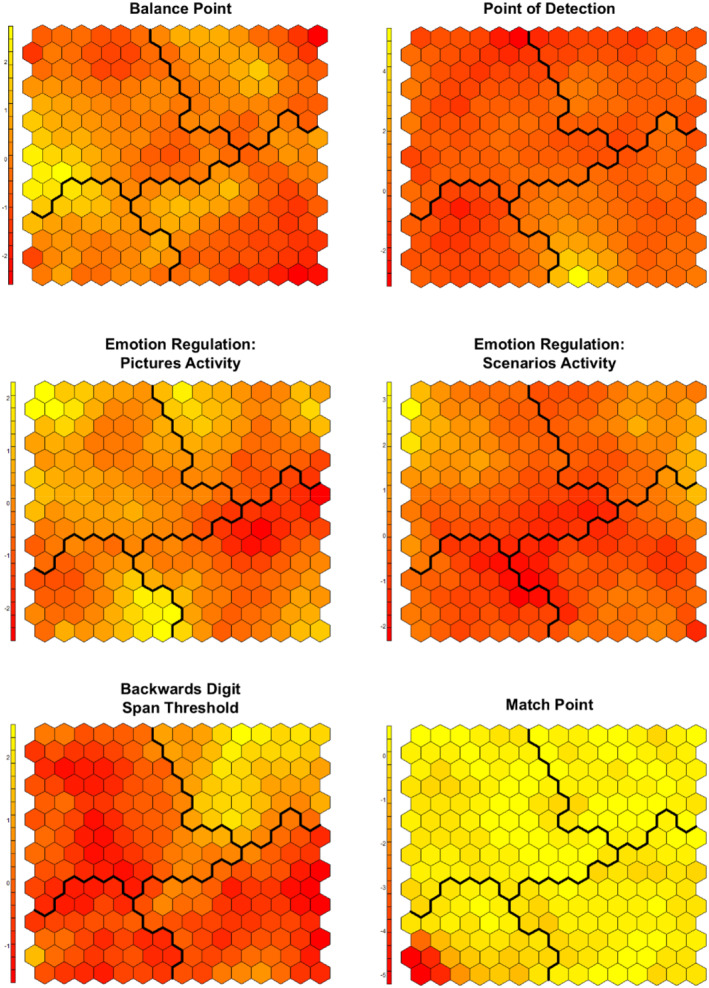
Nodal weight distributions from the SOMs, split by task measure. For each measure, hexagons represent weights of that measure, with red hexagons representing low weights and yellow hexagons representing high weights. Boundaries depicted by the black line indicate borders between the data‐driven clusters applied to the SOM. SOMs = self‐organising maps.

Results of the data‐driven cluster analysis suggested that the questionnaire measures were best captured by a three‐cluster solution, whereas the task‐based measures were best captured by a four‐cluster solution. Although these clusters were overlapping, membership was nonetheless reliable for most clusters. Figures 1 and 2 show the distribution of scores on each measure used to train the SOM, split by cluster. Descriptive statistics, comparisons of missing data between demographic variables, and correlations between measures used in the SOMs can be found Supporting Information S1: Tables S2–S6. Notably, there was no association between cluster membership for the SOM trained on the questionnaire measures and the SOM trained on the task‐based measures (*Χ* ^2^ [6, *N* = 559] = 8.05, *p* = .235). Bootstrap analysis demonstrated that cluster membership was generally stable for both the questionnaire‐based and task‐based cluster solutions and that these were robust to the exclusion of measures with low internal consistency (see Supporting Information S1: Appendix S2).

### Characterising clusters

**FIGURE 3:**
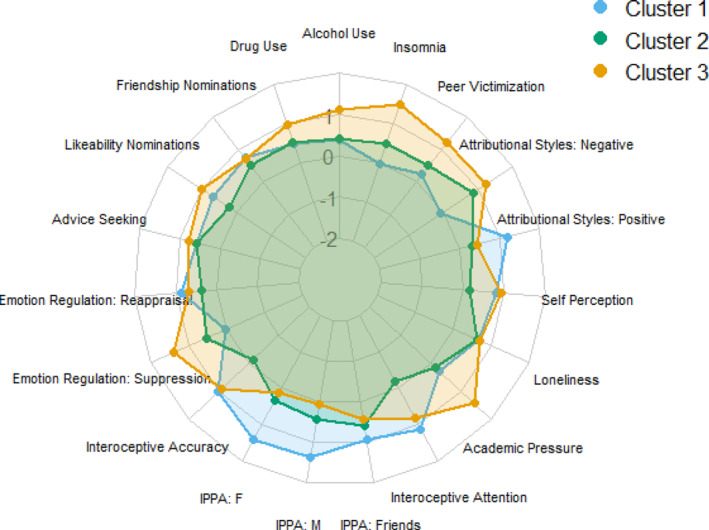
Radar plot showing differences in mean scores on each socio‐affective questionnaire measure between clusters. All *p* values are corrected for FDR. FDR = false discovery rates; IPPA: F = Inventory of Parent and Peer Attachment: Female Caregiver, IPPA: Friends = Inventory of Parent and Peer Attachment: Friends, IPPA: M = Inventory of Parent and Peer Attachment: Male Caregiver.

The questionnaire clusters captured reliable profiles that were differentiated by patterns of responses on the socio‐affective measures (see Figure 3). Cluster 1 (*N* = 203) was defined by having more self‐reported positive relationships with peers and caregivers, making more positive attributions and reporting reduced emotion suppression and insomnia, which we termed the ‘low socio‐affective difficulties’ cluster. Cluster 2 (*N* = 151) was defined by poor self‐reported interoceptive ability, a lower perception of own attributes (i.e., how nice, popular or mature one is relative to peers) and fewer social network nominations from peers, which we termed the ‘disconnected’ cluster (see Table 2 for pairwise comparisons between clusters). Finally, Cluster 3 (*N* = 205) captured profiles of increased self‐reported substance use, greater rates of self‐reported insomnia and academic pressure, greater self‐reported emotion suppression and negative attributions, as well as higher rates of peer victimisation. This final cluster was termed the ‘pervasive socio‐affective difficulties’ cluster.

**TABLE 2:** Table demonstrating the pairwise comparisons between the low socio‐affective difficulties cluster (Cluster 1), disconnected cluster (Cluster 2), and pervasive socio‐affective difficulties cluster (Cluster 3).

Variable	FDR corrected *p* value for comparison: Clusters 1 and 2	FDR corrected *p* value for comparison: Clusters 1 and 3	FDR corrected *p* value for comparison: Clusters 2 and 3
Academic pressure	0.27	<.001	<.001
Attributional styles: Positive	<.001	<.001	.380
Attributional styles: Negative	<.001	<.001	.003
Emotion regulation: Reappraisal	<.001	.190	.054
Emotion regulation: Suppression	<.001	<.001	<.001
Interoceptive attention	<.001	.012	<.001
Interoceptive accuracy	<.001	.400	<.001
Self perception	<.001	.350	<.001
Advice seeking	.980	.370	.370
Alcohol use	.600	<.001	<.001
Drug use	.450	.004	.005
Friendship nominations	.150	.960	.150
Likeability nominations	<.001	.024	<.001
IPPA: Female caregiver	<.001	<.001	.170
IPPA: Male caregiver	<.001	<.001	.012
IPPA: Friends	.024	<.001	.260
Insomnia	<.001	<.001	<.001
Loneliness	.820	.820	.820
Peer victimisation	0.053	<.001	<.001

Abbreviation: FDR = false discovery rates.

**FIGURE 4:**
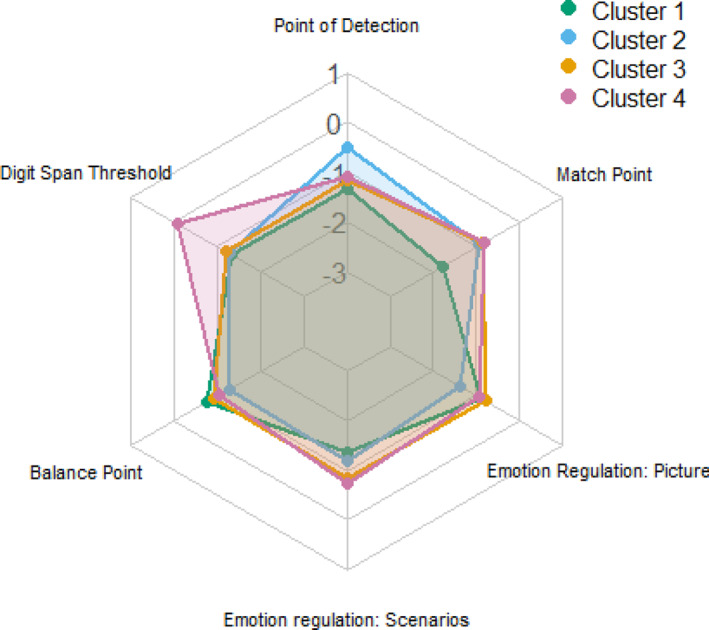
Radar plot showing differences in mean scores on each socio‐affective task measure between clusters. All *p* values are corrected for FDR. The match point and balance point were derived from the interpretation bias task, indexing participants' bias for hostile attributions. The point of detection as derived from the emotional intensity morphing task, measuring sensitivity towards perceiving emotions. Digit span threshold was derived from the backwards digit span task indexing working memory capacity. FDR = false discovery rates.

Differences between the task‐based clusters were less conceptually clear on scores that defined their socio‐affective functioning. For example, Cluster 1 (*N* = 84) which we termed the ‘blunted emotional sensitivity—lower cognitive ability’ cluster appeared to be defined statistically by fewer hostile attributions (a higher balance point) but less consistency in these attributions (a lower match point), as well as lower working memory capacity (a lower digit span threshold; see Figure 4). Cluster 2 (*N* = 149), which we termed the ‘heightened emotional sensitivity—lower cognitive ability’ cluster, was defined by less sensitivity towards recognising emotions (a higher point of detection) more hostile attributions (a lower balance point), lower working memory capacity (a lower digit span threshold) and poorer emotion regulation. Cluster 3 (*N* = 209), the ‘competent emotion processing—lower cognitive ability’ cluster was defined by relatively good emotion regulation (greater ability to downregulate negative emotions) and relative consistency in emotion attributions (a higher match point), but markedly lower working memory capacity (a lower digit span threshold; for pairwise comparisons between clusters, see Table 3). Finally, Cluster 4 (*N* = 117), the ‘competent emotion processing—higher cognitive ability’ cluster, was defined by a competent emotion regulation (greater ability to downregulate negative emotions), relative consistency in emotion attributions (a higher match point), and higher working memory capacity (a higher digit span).

**TABLE 3:** Pairwise comparisons between the four task clusters on the input measures.

Variable	FDR corrected *p* value for comparison: Clusters 1 and 2	FDR corrected *p* value for comparison: Clusters 1 and 3	FDR corrected *p* value for comparison: Clusters 1 and 4	FDR corrected *p* value for comparison: Clusters 2 and 3	FDR corrected *p* value for comparison: Clusters 2 and 4	FDR corrected *p* value for comparison: Clusters 3 and 4
Point of detection	<.001	.057	.004	<.001	<.001	.110
Digit span threshold	.580	.200	<.001	.280	<.001	<.001
Balance point	<.001	.058	.003	<.001	.005	.087
Emotion regulation: Scenarios	.023	<.001	<.001	<.001	<.001	.050
Emotion regulation: Pictures	<.001	.280	.580	<.001	<.001	.028
Match point	<.001	<.001	<.001	.025	.004	.310

Abbreviation: FDR = false discovery rates.

Testing the differences between clusters with respect to age, ethnicity, year group, pubertal status, SEND status, and gender, only gender reached statistical significance for the task‐based clusters. The results suggest an overrepresentation of female participants in the low socio‐affective difficulties cluster (*Χ* ^2^ [4, *N* = 559] = 10.99, *p* = .027).

Overall, there was limited evidence that cluster membership was associated with participant demographic characteristics (see Supporting Information S1: Appendix S3). However, there was an association between gender and questionnaire cluster membership (*Χ* ^2^ [4, *N* = 559] = 10.99, *p* = .027), such that there was an overrepresentation of female participants in the low socio‐affective difficulties cluster and an underrepresentation of female participants in pervasive socio‐affective difficulties cluster.

### Cluster membership is associated with transdiagnostic mental health problems

**FIGURE 5:**
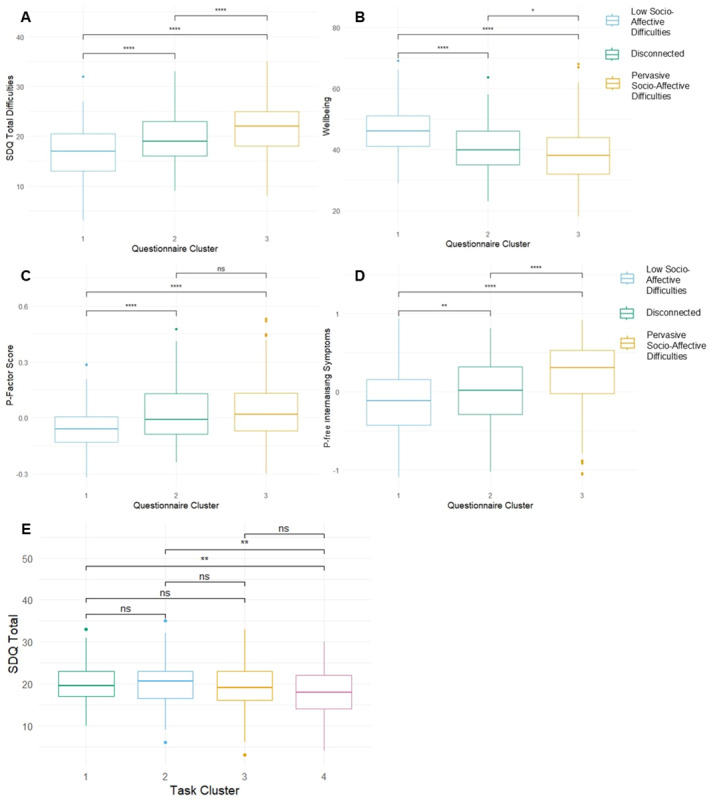
Boxplots demonstrating differences between questionnaire clusters on SDQ total difficulties scores (A), wellbeing (WEMWBS total score; B), p‐factor scores (C) and p‐free internalising factor (D). (E) demonstrates differences between task‐based clusters on SDQ total difficulties scores. SDQ = Strengths and Difficulty Questionnaire. *p < .05, **p < .01, ***p < .001, ****p < .0001.

When we examined whether cluster membership was associated with transdiagnostic mental health outcomes, we found a significant difference in SDQ total difficulties scores between the three questionnaire clusters (*F*(2, 556) = 50.69, *p* < 0.001, *p*_fdr_ < .001, *η* ^2^ = 0.15). Post‐hoc tests demonstrated that those in the pervasive socio‐affective difficulties cluster had higher SDQ total difficulties scores than those in the disconnected (*p*_bonf_ < .001, Cohen's *d* = −0.45, 95% CI [−0.71, −0.19]; Figure 5A) and low socio‐affective difficulties clusters (*p*_bonf_ < .001, Cohen's *d* = −1.00, 95% CI [−1.24, −0.75]), and those in the disconnected cluster scored higher than those in the low socio‐affective difficulties cluster (*p*_bonf_ < .001, Cohen's *d* = 0.54, 95% CI [0.28, 0.80]). Similarly, there were significant differences in wellbeing scores between the three questionnaire clusters (*F*(2, 556) = 49.09, *p* < 0.001, *p*_fdr_ < .001, *η* ^2^ = 0.15; Figure 5B). Post‐hoc tests demonstrated that the cluster with low socio‐affective difficulties reported higher wellbeing scores on WEMWBS than the disconnected cluster (*p*_bonf_ < .001, Cohen's *d* = −0.69, 95% CI [−0.95, −0.43]) and pervasive socio‐affective difficulties cluster (*p*_bonf_ < .001, Cohen's *d* = 0.96, 95% CI [0.71, 1.21]); the disconnected cluster and the pervasive socio‐affective difficulties cluster also differed from each other, with the latter reporting lowest wellbeing (*p*_bonf_ = .036, Cohen's *d* = 0.27, 95% CI [0.01, 0.53]).

There were significant differences on p‐factor scores between questionnaire clusters (*F*(2, 556) = 26.08, *p* < .001, *p*_fdr_ < .001, *η* ^2^ = 0.09). Post‐hoc tests demonstrated that those in the pervasive socio‐affective difficulties cluster had higher p‐factor scores than those in the low socio‐affective difficulties cluster (*p*_bonf_ < .001, Cohen's *d* = −0.68, 95% CI [−0.92, −0.44]; Figure 5C), and those in the disconnected cluster scored higher than those in the low socio‐affective difficulties cluster (*p*_bonf_ < .001, Cohen's *d* = 0.55, 95% CI [0.30, 0.81]). However, there was not a significant difference between he disconnected cluster and the pervasive socio‐affective difficulties cluster on p‐factor scores (*p*_bonf_ = .705). Further, there were significant differences in p‐free internalising factor scores between the questionnaire clusters (*F*(2, 556) = 38.91, *p* < .001, *p*_fdr_ < .001, *η* ^2^ = 0.12). Post‐hoc tests demonstrated that the cluster with pervasive socio‐affective difficulties reported higher p‐free internalising symptoms than the low socio‐affective difficulties cluster (*p*_bonf_ < .001, Cohen's *d* = −0.87, 95% CI [−1.10, −0.63]; Figure 5D) and the disconnected cluster (*p*_bonf_ < .001, Cohen's *d* = −0.55, 95% CI [−0.81, −0.29]); the disconnected cluster and the low socio‐affective difficulties cluster also differed from each other, with the latter reporting lower p‐free internalising symptoms (*p*_bonf_ = .010; Cohen's *d* = 0.32, 95% CI [0.06, 0.58]). There was no significant difference on p‐free externalising symptoms between the questionnaire clusters (*F*(2, 556) = 1.59, *p* = .206).

The only transdiagnostic outcome associated with task cluster membership was SDQ total difficulties (*F*(3, 555) = 3.42, *p* = .017, *η* ^2^ = 0.02), though this did not survive correction for multiple comparisons (*p*_fdr_ = .085). Post‐hoc tests revealed that differences in SDQ total difficulties score were driven by differences between Clusters 1 and 4 (*p*_bonf_ = .048, Cohen's *d* = 0.38, 95% CI [0.01, 0.76]) and Clusters 2 and 4 (*p*_bonf_ = .032, Cohen's *d* = 0.35, 95% CI [0.02, 0.67]; Figure 5E). There were no significant differences between task clusters on wellbeing (*F*(3, 555) = 0.69, *p* = .559, *p*_fdr_ = .559), p‐factor scores (*F*(3, 555) = 1.57, *p* = .196, *p*_fdr_ = .490), p‐free internalising symptoms (*F*(3, 555) = 0.87, *p* = .456, *p*_fdr_ = .559), or p‐free externalising symptoms (*F*(3, 555) = 1.13, *p* = .338, *p*_fdr_ = .559).

## DISCUSSION

Transdiagnostic approach to the study of psychopathology is gaining increasing traction and has the potential to increase our understanding of how mental health risk operates. However, there has been limited examination of how socio‐affective domains/processes cluster and how these are associated with the severity of mental health symptoms. Addressing this gap, this study employed a data‐driven approach to identify clusters of socio‐affective functioning using a battery of questionnaire measures and task‐based measures—focusing on a sample of adolescents at risk of mental health problems. The clusters derived from the questionnaires identified groupings at different levels of symptom severity, but did not align with traditional diagnostic boundaries. These data highlight the role played by different socio‐affective domains in risk for mental health problems, which may therefore be prime candidates for intervention.

The clusters derived from questionnaire measures suggest that problems with emotion functioning and social relationships co‐occur, rather than exist in isolation. For example, the cluster with pervasive socio‐affective difficulties demonstrated higher rates of maladaptive emotion regulation strategies, such as rumination, as well as reporting higher rates of peer victimisation. Although these findings cannot disentangle the bidirectional association between emotion processing and social problems, they are consistent with previous longitudinal work demonstrating that these processes can negatively affect one another, with consequences for mental health (Demkowicz et al., 2024). The findings also underline the value of adopting a person‐centred, rather than variable‐centred, approach to characterising adolescent functioning.

The clusters of socio‐affective functioning derived from questionnaire data demonstrated that there are profiles of socio‐affective functioning that are associated with more severe symptoms of mental health problems in adolescence. For example, the pervasive socio‐affective difficulties cluster scored higher than the disconnected and low socio‐affective difficulties clusters on overall psychopathology, measured using the SDQ, factors score for p‐free internalising problems, and wellbeing. Moreover, both the pervasive socio‐affective difficulties and disconnected clusters scored higher than the low socio‐affective difficulties cluster on the mental health outcomes. The sample of adolescents in this study all exhibited elevated rates of mental health problems and the degree of severity seemed to track degree of socio‐affective difficulties. In the future, it will be important to examine whether measurement of socio‐affective difficulties, alongside symptom screening, may add to the understanding of whose mental health problems are most persistent.

These findings are consistent with evidence that mental health problems are multicausal, with both social and emotional problems contributing to mental health risk (Kendler, 2019). These converging lines of evidence suggest that transdiagnostic interventions for mental health risk should address both domains and consider the potential transactional nature of social and emotional difficulties. This study also represents an effort to use task‐based measures to identify social and cognitive processes that might be targeted in interventions for young people (Wolpert et al., 2021). There are well documented challenges with cluster analyses using task‐based data (Gao et al., 2023), which may have affected the discriminability of the task‐based clusters. For example, issues with poor reliability (Pezzoli et al., 2023) and outliers (Gao et al., 2023) can both affect clustering algorithms. Indeed, task‐based measures are susceptible to state effects (e.g., mood, fatigue; Eldar & Niv, 2015; Shigihara et al., 2013), which introduce additional noise that can hinder clustering algorithms. Despite these challenges, we observed different clusters of functioning using task‐based measures, which is an advantage of using SOMs over other approaches as they can account for non‐linear relationships between input measures (Hulle, 2000). Further, there was some tentative evidence that these clusters differed on mental health symptoms, with two clusters associated with higher rates of mental health difficulties. These clusters did not differ from each other on their levels of symptoms, but differed in their cognitive and affective risk mechanism profile. This may indicate heterogeneity within adolescents at risk and highlights that different adolescents may require partially different approaches. Of course, the tentative and cross‐sectional nature of these data must be emphasised, and further work testing alternative approaches to estimating latent factors of psychopathology (e.g., a two‐factor model only estimating externalising and internalising factors) might yield further nuanced insights. It is also important to note that task‐based behaviour is adaptive based on context. For example, a bias to perceive emotion as hostile can be adaptive in circumstances of high threat (e.g., children exposed to physical abuse). Such context‐specific adaption is not accounted for in clustering solutions and is an area that also warrants further consideration.

There was a notable absence of an association between questionnaire and task cluster membership, which may suggest that these clusters capture different risk factors for poor mental health (Friedman & Banich, 2019). However, it is also plausible that the different indicators used to index these processes contributed to the different cluster solutions. For example, the emotion regulation questionnaire measured the use of specific emotion regulation strategies, whereas the emotion regulation tasks assessed a general ability to regulate negative affect. Nevertheless, the distinction between questionnaire and task‐based cluster membership is also consistent with previous research, where clusters have been characterised by predominantly parent ratings‐based or task‐based profiles, suggesting these measures capture distinct processes (Mareva et al., 2023, 2024). Taken together, these findings should motivate more work on testing the utility of different measures for understanding transdiagnostic mental health risk and how this may vary depending on populations or contexts. One such way to examine how context might affect the utility of task‐based measures in predicting mental health risk would be to examine how performance on a wider array of tasks is moderated by variables such as insomnia and substance use.

Although there was limited evidence of demographic characteristics that distinguished the questionnaire and task‐based clusters, this is unsurprising considering participants all had elevated mental health risk, were recruited from schools with similar characteristics and from a narrow cohort within participating schools. Previous research adopting similar methods has recruited participants across a larger age range (Mareva et al., 2023, 2024) and it is likely that age‐related differences in cluster membership emerge as socio‐affective processes develop over adolescence and into young adulthood (Silvers, 2022). Given evidence that cluster membership can change across development (Benhamou et al., 2025), it would be important to examine whether those moving from clusters defined by pervasive socio‐affective difficulties to clusters with fewer socio‐affective difficulties exhibit a decrease in mental health symptoms. Such work would also inform the extent to which these methods (i.e., unsupervised machine learning and cluster analyses) might have clinical utility.

There are limitations with this study that are important to note. The sample in this study were recruited based on a criterion of heightened rates of psychopathology. This sampling strategy may have restricted the ability of the clusters to distinguish rates of mental health problems, as symptoms were negatively skewed in this population. The sample recruited for this study may have also reduced the variability in the measures used to train the SOM, affecting the final number and distribution of clusters derived from the data. Future work should replicate these findings in a sample with greater variability in mental health symptoms to examine whether the same socio‐affective profiles are identified, and whether these have improved sensitivity in detecting mental health symptoms. In addition, it should be noted that some of the measures used to train the SOM (specifically the attributional styles subscales) had relatively poor internal consistency, which can produce unstable cluster solutions. To address this issue, we conducted sensitivity analyses excluding these measures and found cluster assignment was robust to the exclusion of these measures (see Supporting Information S1: Appendix S2). Finally, the silhouette scores for the clusters were relatively low, which indicates limited differentiability between clusters. Rather, these data may instead reflect overlapping distributions that we have distinguished between, with many boundary cases. However, the clusters demonstrated different profiles of socio‐affective functioning and health symptomology. These findings may therefore have value when considering person‐centred approaches to transdiagnostic risk factors for psychopathology in adolescence, but require replication and further validation before they might be considered for any clinical utility.

In conclusion, this study has demonstrated the potential of data‐driven methods to derive profiles of socio‐affective functioning that are associated with a range of mental health problems. These findings contribute to the literature on transdiagnostic approaches by identifying socio‐affective domains that are associated with heightened rates of psychopathology and may therefore present viable targets for future interventions. As the clusters with highest rates of mental health problems exhibited a combination of social and emotional problems, it will be important to examine whether targeting these processes in tandem may be an effective strategy to effectively reduce transdiagnostic mental health problems. Indeed, mechanistic trials (i.e., those that measure mechanistic processes in addition to key mental health outcomes) might be well‐suited to testing such a hypothesis. Although findings from the task‐based clusters were less conceptually clear, they encourage future task development to enable more sensitive and comprehensive investigations. Data from this study tentatively suggest that whilst questionnaire‐based measures may identify clusters that vary in severity and number of domains affected, task‐based measures may help identify different socio‐affective profiles of groups who display similar kinds and severity of symptoms. Task based measures may therefore offer more precise insights of the types of mechanisms that should be targeted for different adolescents at risk. Overall, these findings contribute to better understanding transdiagnostic risk factors for psychopathology and contribute to efforts to identify socio‐affective mechanisms that, if targeted for intervention, may improve outcomes for young people at risk of mental health problems.

## AUTHOR CONTRIBUTIONS

**Alex Lloyd**: Conceptualization; investigation; writing—original draft; methodology; visualization; formal analysis; data curation. **Duncan Astle**: Conceptualization; writing—review and editing; methodology; supervision. **Tom (Chin‐Han) Wu**: Data curation; writing—review and editing. **Nikolaus Steinbeis**: Supervision; writing—review and editing; funding acquisition. **Ritika Chokhani**: Formal analysis; writing—review and editing; methodology. **Laura Lucas**: Project administration; data curation; writing—review and editing. **Pasco Fearon**: Conceptualization; investigation; funding acquisition; writing—review and editing; supervision; **Essi Viding**: Conceptualization; investigation; funding acquisition; writing—original draft; writing—review and editing; supervision.

## CONFLICT OF INTEREST STATEMENT

The authors declare no conflicts of interest.

## ETHICAL CONSIDERATIONS

Informed consent was obtained from all participants and their parents/carers prior to taking part in the study. Ethical approval was received from UCL's Research Ethics Committee (ref: 21815/001, approved on 22/02/2022).

## Supporting information

Supporting Information S1

## Data Availability

The data that support the findings of this study are available from the corresponding author upon reasonable request. Data will be made publicly available 12 months after the project end (project end date: March 2026).
