# Supplementary material for: Transdiagnostic profiles of socio‐affective functioning in adolescents at‐risk of poor mental health
Source: JCPP Adv. 2026 Jun 22:e70137. Online ahead of print. doi: 10.1002/jcv2.70137 (PMC13339588; doi:10.1002/jcv2.70137)
Supplement: Supplementary file 1 — Supporting Information S1 [file JCV2-9999-e70137-s001.docx]

**Transdiagnostic Profiles of Socio-Affective Functioning in Adolescents**

**At-Risk of Poor Mental Health**

**Supporting Information**

**Appendix S1. Model Parameters and Fit Statistics for P-Factor, P-free Internalizing and P-free Externalizing Factors**

As specified in the Statistical Analysis Plan for the trial from which these data were derived, a confirmatory factor analysis (CFA) of all available data was fit to the SDQ and Me and My Feelings measures to estimate the p-factor, internalising and externalising symptoms. CFA were fit in R and Stata. For the purpose of the CFA, the observed variables were treated as continuous. To further improve the estimates, data from the baseline assessment (reported in the main manuscript) and post-intervention assessment (not reported in the main manuscript) were used. The model fitted moderately well (RMSEA = 0.07 [95 % CI: 0.066; 0.073], CFI = 0.87, TLI = 0.85 SRMR = 0.07). There were no appreciable differences (other than those expected from random variation) between the estimated loadings of this model against the loadings derived from the same model estimated only on the baseline data. Factor scores for the p-factor, p-free internalising and p-free externalising were then estimated from regression factor scores. See Table S6 for estimated factor loadings from the CFA analysis of baseline and end-of-treatment scores.

**Table S1**: estimated factor loadings from the CFA analysis of baseline and end-of-treatment scores.

| Variable | General Psychopathology | P-free Internalising | P-free Externalising |
| --- | --- | --- | --- |
| sdq_3 | 0.23 | 0.38 |  |
| sdq_8 | -0.04 | 0.75 |  |
| sdq_13 | 0.32 | 0.66 |  |
| sdq_16 | -0.05 | 0.53 |  |
| sdq_24 | -0.01 | 0.53 |  |
| sdq_5 | 0.26 |  | 0.72 |
| sdq_7 | 0.39 |  | 0.12 |
| sdq_12 | 0.51 |  | 0.27 |
| sdq_18 | 0.45 |  | 0.15 |
| sdq_22 | 0.42 |  | -0.03 |
| mmf_1 | 0.25 | 0.57 |  |
| mmf_2 | 0.20 | 0.60 |  |
| mmf_3 | 0.31 | 0.62 |  |
| mmf_4 | 0.27 | 0.51 |  |
| mmf_5 | -0.04 | 0.81 |  |
| mmf_6 | 0.32 | 0.33 |  |
| mmf_7 | 0.30 | 0.29 |  |
| mmf_9 | 0.12 | 0.64 |  |
| mmf_10 | 0.10 | 0.73 |  |
| mmf_11 | 0.31 |  | 0.78 |
| mmf_12 | 0.28 |  | 0.87 |
| mmf_13 | 0.52 |  | 0.51 |
| mmf_14 | 0.59 |  | 0.26 |
| mmf_15 | 0.23 |  | 0.23 |
| mmf_16 | 0.53 |  | 0.15 |

*sdq: Strengths and difficulties questionnaire; mmf: Me and My Feelings Questionnaire*

Four of the 14 internalizing items had low loadings onto the general psychopathology factor. We inspected these four items, which referred to cognitive-emotional features of anxiety (‘I worry a lot’ (items on both the SDQ and Me and My Feelings), ‘I am nervous in new situation. I easily lose confidence’ and ‘I have many fears, I am easily scared’). To examine whether the general psychopathology factor was able to capture transdiagnostic dimensions, including anxiety, we examined the association between the general psychopathology factor and scores on the GAD-7. This analysis demonstrated there was a modest positive correlation between the general psychopathology factor and GAD-7 (*r*=0.35, *p* < .001), suggesting the factor measured general psychopathology despite the low loading of some items.

Table S2: Mean and SD of measures used to train the SOMs after multiple imputation, but prior to z-scoring.

|  |  | **Mean** | **SD** |
| --- | --- | --- | --- |
|  | *Questionnaire Measures* |  |  |
|  | Friendship nomination (indegree) | 2.35 | 1.81 |
|  | Likeability nomination (indegree) | 1.92 | 1.89 |
|  | Advice seeking (outdegree) | 3.94 | 2.35 |
|  | Emotion regulation: Reappraisal | 17.89 | 4.49 |
|  | Emotion regulation: Suppression | 13.24 | 2.98 |
|  | Interoceptive Accuracy | 74.63 | 12.49 |
|  | Caregiver relationship (female caregiver) | 89.09 | 20.18 |
|  | Caregiver relationship (male caregiver) | 86.23 | 21.76 |
|  | IPPA: Friendships | 88.60 | 15.67 |
|  | Interoceptive Attention | 73.22 | 13.68 |
|  | Academic Pressure | 21.46 | 5.35 |
|  | Loneliness | 10.01 | 1.33 |
|  | Self-perception relative to peers | 9.58 | 2.22 |
|  | Attributional styles: Positive attributions | 6.02 | 2.25 |
|  | Attributional styles: Negative attributions | 5.46 | 2.24 |
|  | Peer victimization | 42.25 | 11.78 |
|  | AUDIT | 0.11 | 0.38 |
|  | DUDIT | 0.06 | 0.35 |
|  | Insomnia | 0 (mode) | - |
|  | *Task-Based Measures* |  |  |
|  | Emotion perception: Point of detection | 7.75 | 0.86 |
|  | Backward Digit Span Threshold | 4.50 | 1.00 |
|  | Interpretation Bias Task: Balance point | 7.47 | 1.64 |
|  | Interpretation Bias Task: Match point | 39.02 | 6.43 |
|  | Emotion regulation: Scenarios task | 0.19 | 0.68 |
|  | Emotion regulation: Pictures task | 0.17 | 0.77 |

Table S3: Testing rates of missingness between gender.

| Variable | Chi Square Test statistic | Gender overrepresented (if applicable) |
| --- | --- | --- |
| Phase Adjustment Task (status) | *Χ^2^* = 13.40, *p* = .009 | Female |
| Academic pressure | *Χ^2^* = 3.10 *p* = .268* |  |
| Friendship indegree | *Χ^2^* = 0.50, *p* = .974 |  |
| Likeability indegree | *Χ^2^* = 0.50, *p* = .974 |  |
| Advice outdegree | *Χ^2^* = 0.50, *p* = .974 |  |
| Self-perception | *Χ^2^* = 2.88, *p* = .577 |  |
| Interoceptive attention | *Χ^2^* = 4.07, *p* = .302* |  |
| Peer victimization | *Χ^2^* = 0.20, *p* = .999* |  |
| Balance point | *Χ^2^* = 0.12, *p* = .999* |  |
| Interoceptive accuracy | *Χ^2^* = 0.95, *p* = .470 |  |
| IPPA: Friends | *Χ^2^* = 111.20, *p* = .016* | Male and female overrepresented relative to those not reporting gender |
| Loneliness | *Χ^2^* = 0.12, *p* = .999* |  |
| Total positive attributions | *Χ^2^* = 0.14, *p* = .999* |  |
| Total negative attributions | *Χ^2^* = 0.14, *p* = .999* |  |
| Point of detection | *Χ^2^* = 3.09, *p* = .204* |  |
| Emotion regulation: Reappraisal | *Χ^2^* = 0.13, *p* = .999* |  |
| Emotion regulation: Suppression | *Χ^2^* = 0.13, *p* = .999* |  |
| Emotion regulation task: Decrease | *Χ^2^* = 0.72, *p* = .999* |  |
| Emotion regulation task: Look | *Χ^2^* = 0.72, *p* = .999* |  |

* Fisher test corrected

Table S4: Testing rates of missingness between year groups.

| Variable | Chi Square Test statistic | Year group overrepresented (if applicable) |
| --- | --- | --- |
| Phase Adjustment Task (status) | *Χ^2^* = 0.27, *p* = .605 |  |
| Academic pressure | *Χ^2^* = 0.01 *p* = .724* |  |
| Friendship indegree | *Χ^2^* = 0.01, *p* = .999 |  |
| Likeability indegree | *Χ^2^* = 0.01, *p* = .999 |  |
| Advice outdegree | *Χ^2^* = 0.01, *p* = .999 |  |
| Self-perception | *Χ^2^* = 0.56, *p* = .450 |  |
| Interoceptive attention | *Χ^2^* = 0.01, *p* = .999 |  |
| Peer victimization | *Χ^2^* = 0.58, *p* = .450* |  |
| Balance point | *Χ^2^* = 0.83, *p* = .215* |  |
| Interoceptive accuracy | *Χ^2^* = 0.01, *p* = .999* |  |
| IPPA: Friends | *Χ^2^* = 0.79, *p* = .373* |  |
| Loneliness | *Χ^2^* = 0.01, *p* = .999* |  |
| Total positive attributions | *Χ^2^* = 0.01, *p* = .999* |  |
| Total negative attributions | *Χ^2^* = 0.01, *p* = .999* |  |
| Point of detection | *Χ^2^* = 0.01, *p* = .999* |  |
| Emotion regulation: Reappraisal | *Χ^2^* = 0.01, *p* = .999* |  |
| Emotion regulation: Suppression | *Χ^2^* = 0.01, *p* = .999* |  |
| Emotion regulation task: Decrease | *Χ^2^* = 0.01, *p* = .450* |  |
| Emotion regulation task: Look | *Χ^2^* = 0.01, *p* = .450* |  |

* Fisher test corrected

Table S5: Correlations between the task-based measures with confidence intervals.

| *Variable* | *1* | *2* | *3* | *4* | *5* |
| --- | --- | --- | --- | --- | --- |
|  |  |  |  |  |  |
| *1.* Emotion perception: Point of detection |  |  |  |  |  |
|  |  |  |  |  |  |
| *2.* Backwards Digit Span | *-.05* |  |  |  |  |
|  | *[-.13, .04]* |  |  |  |  |
|  |  |  |  |  |  |
| *3.* Interpretation Bias Task: Balance point | *-.07* | *-.05* |  |  |  |
|  | *[-.15, .02]* | *[-.13, .03]* |  |  |  |
|  |  |  |  |  |  |
| *4.* Emotion regulation: Scenarios task | *-.06* | *.10** | *-.06* |  |  |
|  | *[-.15, .02]* | *[.01, .18]* | *[-.14, .02]* |  |  |
|  |  |  |  |  |  |
| *5.* Emotion regulation: Pictures Task | *-.01* | *-.01* | *-.07* | *.07* |  |
|  | *[-.09, .07]* | *[-.09, .08]* | *[-.16, .01]* | *[-.02, .15]* |  |
|  |  |  |  |  |  |
| *6. Interpretation Bias Task: Match Point* | *.03* | *.04* | *.01* | *.01* | *.02* |
|  | *[-.05, .11]* | *[-.04, .12]* | *[-.07, .10]* | *[-.08, .09]* | *[-.07, .10]* |
|  |  |  |  |  |  |

*M* and *SD* are used to represent mean and standard deviation, respectively. Values in square brackets indicate the 95% confidence interval for each correlation. The confidence interval is a plausible range of population correlations that could have caused the sample correlation (Cumming, 2014). * indicates *p* < .05. ** indicates *p* < .01.

Table S6: Correlations between the questionnaire measures with confidence intervals.

| Variable | 1 | 2 | 3 | 4 | 5 | 6 | 7 | 8 | 9 | 10 | 11 | 12 | 13 | 14 | 15 |
| --- | --- | --- | --- | --- | --- | --- | --- | --- | --- | --- | --- | --- | --- | --- | --- |
|  |  |  |  |  |  |  |  |  |  |  |  |  |  |  |  |
| 1. Friendship indegree |  |  |  |  |  |  |  |  |  |  |  |  |  |  |  |
|  |  |  |  |  |  |  |  |  |  |  |  |  |  |  |  |
| 2. Likability in degree | .61** |  |  |  |  |  |  |  |  |  |  |  |  |  |  |
|  | [.55, .66] |  |  |  |  |  |  |  |  |  |  |  |  |  |  |
|  |  |  |  |  |  |  |  |  |  |  |  |  |  |  |  |
| 3. Advice seeking outdegree | .18** | .14** |  |  |  |  |  |  |  |  |  |  |  |  |  |
|  | [.09, .26] | [.06, .22] |  |  |  |  |  |  |  |  |  |  |  |  |  |
|  |  |  |  |  |  |  |  |  |  |  |  |  |  |  |  |
| 4. Emotion regulation: Reappraisal | .02 | .07 | .03 |  |  |  |  |  |  |  |  |  |  |  |  |
|  | [-.06, .10] | [-.01, .16] | [-.05, .11] |  |  |  |  |  |  |  |  |  |  |  |  |
|  |  |  |  |  |  |  |  |  |  |  |  |  |  |  |  |
| 5. Emotion regulation: Suppression | -.01 | .05 | -.00 | .10* |  |  |  |  |  |  |  |  |  |  |  |
|  | [-.09, .07] | [-.03, .14] | [-.08, .08] | [.02, .18] |  |  |  |  |  |  |  |  |  |  |  |
|  |  |  |  |  |  |  |  |  |  |  |  |  |  |  |  |
| 6. Interoceptive accuracy | .11* | .09* | .06 | .05 | -.07 |  |  |  |  |  |  |  |  |  |  |
|  | [.03, .19] | [.01, .17] | [-.02, .14] | [-.04, .13] | [-.15, .02] |  |  |  |  |  |  |  |  |  |  |
|  |  |  |  |  |  |  |  |  |  |  |  |  |  |  |  |
| 7. IPPA female caregiver | .02 | -.07 | .09* | .23** | -.27** | .04 |  |  |  |  |  |  |  |  |  |
|  | [-.07, .10] | [-.16, .02] | [.00, .18] | [.15, .32] | [-.35, -.18] | [-.05, .13] |  |  |  |  |  |  |  |  |  |
|  |  |  |  |  |  |  |  |  |  |  |  |  |  |  |  |
| 8. IPPA male caregiver | .02 | -.02 | .10* | .26** | -.25** | .01 | .63** |  |  |  |  |  |  |  |  |
|  | [-.07, .11] | [-.11, .07] | [.01, .19] | [.17, .34] | [-.33, -.16] | [-.08, .10] | [.57, .68] |  |  |  |  |  |  |  |  |
|  |  |  |  |  |  |  |  |  |  |  |  |  |  |  |  |
| 9. IPPA Friendships | .15** | .08 | .13** | .20** | -.11** | .11** | .29** | .32** |  |  |  |  |  |  |  |
|  | [.07, .24] | [-.00, .16] | [.05, .21] | [.12, .28] | [-.20, -.03] | [.03, .19] | [.21, .37] | [.24, .40] |  |  |  |  |  |  |  |
|  |  |  |  |  |  |  |  |  |  |  |  |  |  |  |  |
| 10. Interoceptive attention | .09* | .07 | .05 | .14** | -.01 | .59** | .14** | .17** | .15** |  |  |  |  |  |  |
|  | [.00, .17] | [-.01, .16] | [-.04, .13] | [.05, .22] | [-.09, .07] | [.53, .64] | [.05, .22] | [.08, .26] | [.06, .23] |  |  |  |  |  |  |
|  |  |  |  |  |  |  |  |  |  |  |  |  |  |  |  |
| 11. Academic Pressure | .08 | .15** | .00 | .00 | .24** | .20** | -.24** | -.25** | -.07 | .24** |  |  |  |  |  |
|  | [-.01, .16] | [.07, .23] | [-.08, .09] | [-.08, .09] | [.16, .32] | [.12, .28] | [-.32, -.16] | [-.33, -.16] | [-.15, .02] | [.16, .32] |  |  |  |  |  |
|  |  |  |  |  |  |  |  |  |  |  |  |  |  |  |  |
| 12. Loneliness | -.00 | -.02 | -.02 | -.04 | -.01 | .00 | -.04 | -.02 | -.13** | -.02 | .05 |  |  |  |  |
|  | [-.09, .08] | [-.10, .07] | [-.11, .06] | [-.12, .04] | [-.10, .07] | [-.08, .09] | [-.13, .05] | [-.11, .07] | [-.21, -.05] | [-.11, .06] | [-.03, .14] |  |  |  |  |
|  |  |  |  |  |  |  |  |  |  |  |  |  |  |  |  |
| 13. Self perception relative to peers | .15** | .14** | .09* | .15** | .03 | .07 | .06 | .12** | .13** | .08 | .05 | -.03 |  |  |  |
|  | [.07, .23] | [.06, .22] | [.00, .17] | [.06, .23] | [-.05, .11] | [-.02, .15] | [-.03, .15] | [.03, .21] | [.05, .22] | [-.00, .17] | [-.03, .13] | [-.12, .05] |  |  |  |
|  |  |  |  |  |  |  |  |  |  |  |  |  |  |  |  |
| 14. Attributional Styles: Positive | .08 | .07 | .12** | .24** | -.19** | .07 | .37** | .36** | .22** | .14** | -.15** | -.01 | .20** |  |  |
|  | [-.00, .16] | [-.01, .16] | [.03, .20] | [.16, .32] | [-.27, -.11] | [-.01, .15] | [.29, .45] | [.27, .43] | [.13, .29] | [.06, .22] | [-.23, -.06] | [-.09, .07] | [.12, .28] |  |  |
|  |  |  |  |  |  |  |  |  |  |  |  |  |  |  |  |
| 15. Attributional styles: Negative | .02 | -.01 | -.05 | -.26** | .24** | -.01 | -.35** | -.39** | -.20** | -.09* | .23** | .00 | -.13** | -.38** |  |
|  | [-.06, .11] | [-.09, .08] | [-.14, .03] | [-.33, -.18] | [.17, .32] | [-.10, .07] | [-.43, -.27] | [-.47, -.31] | [-.28, -.12] | [-.18, -.01] | [.15, .31] | [-.08, .08] | [-.21, -.05] | [-.45, -.31] |  |
|  |  |  |  |  |  |  |  |  |  |  |  |  |  |  |  |
| 16. Peer victimization | .03 | .04 | -.01 | -.06 | .10* | .12** | -.26** | -.23** | -.21** | .11** | .27** | -.04 | -.04 | -.14** | .23** |
|  | [-.05, .12] | [-.05, .12] | [-.10, .07] | [-.14, .03] | [.01, .18] | [.04, .20] | [-.34, -.18] | [-.31, -.14] | [-.28, -.13] | [.03, .19] | [.19, .35] | [-.12, .05] | [-.12, .05] | [-.22, -.06] | [.15, .30] |
|  |  |  |  |  |  |  |  |  |  |  |  |  |  |  |  |

*M* and *SD* are used to represent mean and standard deviation, respectively. Values in square brackets indicate the 95% confidence interval for each correlation. The confidence interval is a plausible range of population correlations that could have caused the sample correlation (Cumming, 2014). * indicates *p* < .05. ** indicates *p* < .01.

**Appendix S2. Sensitivity Analysis Excluding Measures with Low Internal Consistency**

To assess whether the inclusion of measures with low internal consistency (specifically the positive and negative subscales of the attributional styles questionnaire), we conducted sensitivity analyses to examine whether cluster membership was robust to the exclusion of these measures. We reran the SOM and cluster analysis with these measures excluded and examined the association between cluster membership with and without the attributional styles questionnaire. A Chi Square test of association demonstrated a significant relationship X(df=6, N=559) = 252.98, p < .001) between membership of the clusters with and without the attributional styles scales included, suggesting these measures did not introduce unreliability or instability into the cluster solutions.

**Appendix S3. Lack of evidence for associations between cluster classification and demographics**

There was limited evidence for an association between cluster membership and demographic characteristics, including: age (Questionnaire cluster: *F*(2,556) = 2.44, *p* = .088; Task cluster (*F*(3,555) = 2.28, *p* = .078), ethnicity (Questionnaire cluster: *Χ^2^* (36, N = 559) = 39.21, *p* = .328; Task cluster: *Χ^2^* (54, N = 559) = 47.29, *p* = .729), year group (Questionnaire cluster: *Χ^2^* (3, N = 559) = 3.61, *p* = .307; Task cluster: *Χ^2^* (2, N = 559) = 5.36, *p* = .069), pubertal status (Questionnaire cluster: : *F*(2,439) = 1.52, *p* = .220; Task cluster (*F*(3,438) = 1.04, *p* = .373), SEND status (Questionnaire cluster: *Χ^2^* (2, N = 559) = 3.49, *p* = .175; Task cluster: *Χ^2^* (3, N = 559) = 4.88, *p* = .181) or gender for the task cluster (*Χ^2^* (6, N = 559) = 11.93, *p* = .064).
